# Selective Nonoperative Management of Abdominal Stab Wounds in Low‐ and Middle‐Income Countries: A Systematic Review and Meta‐Analysis

**DOI:** 10.1002/wjs.12517

**Published:** 2025-03-17

**Authors:** Samuel Moffatt, Daniel Biggs, Victor Kong, Damian Clarke

**Affiliations:** ^1^ Department of Surgery, University of KwaZulu‐Natal Durban South Africa; ^2^ Department of Vascular Surgery University Hospitals of Leicester NHS Trust Leicester UK; ^3^ Keele University School of Medicine Newcastle‐under‐Lyme UK; ^4^ Department of Surgery University of Auckland Auckland New Zealand; ^5^ Department of Surgery Auckland City Hospital Auckland New Zealand; ^6^ Department of Surgery University of the Witwatersrand Johannesburg South Africa

**Keywords:** abdominal injury, penetrating trauma, stab wounds, trauma

## Abstract

**Background:**

Selective nonoperative management (SNOM) of abdominal stab wounds is a well‐established approach to managing these injuries and has been practiced since the 1960s. This systematic review and meta‐analysis provides an up‐to‐date analysis of the safety and feasibility of this management strategy in upper‐middle‐income, lower‐middle‐income, low‐income, and least developed countries and describes evidence of how this management has evolved.

**Methods:**

Medline (via PubMed), Google Scholar, Scopus, Embase, the International Clinical Trials Registry, and Web of Science were searched for studies describing SNOM of abdominal stab wounds in patients ≥ 16 years old in upper‐middle‐income, lower‐middle‐income, low‐income, and least developed countries. Study characteristics and method of SNOM (use of computed tomography scanning vs. serial examination only) were extracted. Pooled results for failure of SNOM, mortality, complications, and length of stay (LOS) were analyzed.

**Results:**

Twenty studies were selected containing 1505 patients initially managed nonoperatively with 245 ultimately requiring surgical intervention. The pooled risk of failure of SNOM was 0.14 (95% CI = 0.08–0.22). There was zero reported mortality in patients selected for SNOM. LOS was generally higher in patients undergoing primary operative management, and complications were also reported as being more frequent in primary operative management patients.

**Conclusion:**

SNOM of abdominal stab wounds is a safe method of managing these injuries. Computed tomography is now commonly used to aid in decision‐making about suitability for attempted SNOM in lower‐resource settings.

## Graphical abstract

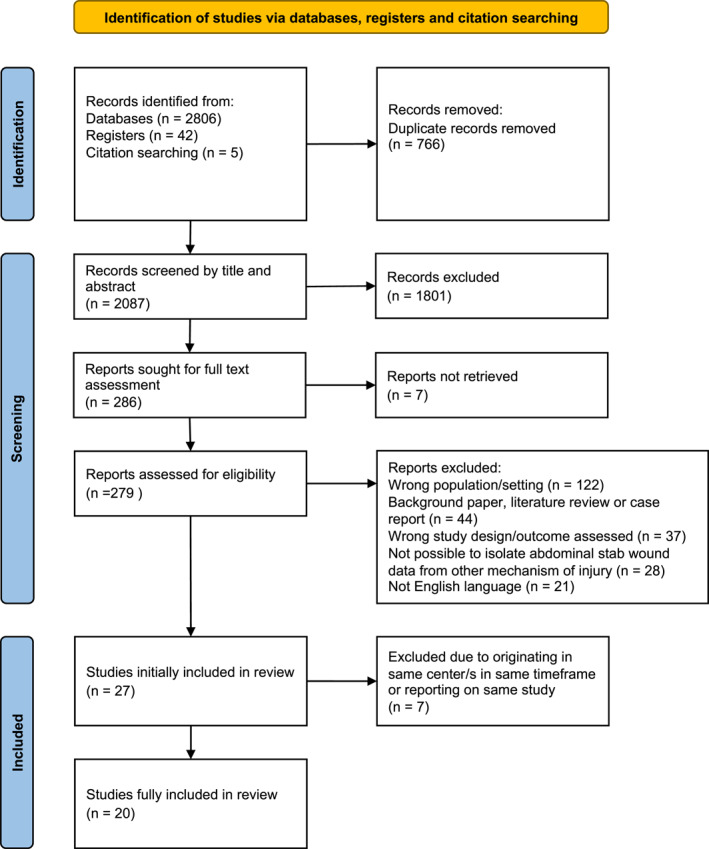

## 1 Introduction

Prior to the 1960s, laparotomy was the standard management approach for all patients who sustained penetrating abdominal trauma. A landmark paper by Shaftan in 1960 in the United States challenged this approach. Shaftan demonstrated that with careful selection and serial clinical observation, it is safe to manage abdominal trauma nonoperatively—with the majority of patients included in his paper suffering abdominal stab wounds (SW) [1]. This initial paper was followed by a number of others that further demonstrated the feasibility of this selective nonoperative management (SNOM) approach [2, 3, 4]. The benefits of SNOM for resource‐limited centers with high penetrating trauma burdens have led to it being adopted not only for abdominal SW but also for gunshot wounds (GSW) [5]. SNOM can reduce the financial cost of managing abdominal SW and has the advantage of reducing the incidence of unnecessary exploration, thus reducing laparotomy associated complications [6]. The crucial aspect of safe SNOM of abdominal SW is patient selection. It is widely accepted that hemodynamically unstable and/or peritonitic patients should be managed with immediate operative management. However, further selection of patients for SNOM is nuanced, with different centers adopting varied approaches. These include the use of diagnostic modalities such as diagnostic peritoneal lavage [7], ultrasonography [8, 9], and computed tomography (CT) [10]—despite SNOM originally being reliant on clinical examination. The intricacies of using these adjuvant diagnostic modalities are also highly variable among centers. Some institutions perform CT with intravenous contrast exclusively, whereas others use triple‐contrast approaches [11].

Although careful observation of the patient during SNOM remains the foundation of the approach, there can be considerable variation in how different centers achieve this. The timing of serial abdominal examination [12, 13], the use/frequency of blood tests [14, 15] (with particular focus on hemoglobin and white cells), and how frequently patient vital signs are observed [15, 16] can be quite variable. Additionally, the optimum observation period before an oral diet is reintroduced and beyond which the conservative approach is considered successful is also highly variable [17]. Factors such as the trauma burden faced by an individual center, the availability of imaging resources (along with the required skill set to employ/interpret those resources), and how easily a patient who has declined during the SNOM observation period can be taken to the theater may guide the individual center’s protocols/approach. The purpose of this systematic review and meta‐analysis was to reexamine the safety of SNOM and provide an update on its use in settings that are likely to have more limited resources and higher trauma burdens (upper‐middle‐income, lower‐middle‐income, low‐income, and least developed countries) and to examine how centers in these settings have incorporated methods such as focused assessment with sonography for trauma (FAST) scan or CT scanning into SNOM of abdominal SW. This is important in evaluating the use of SNOM in these lower‐resource settings but may also offer lessons for higher‐resource settings where the opportunity to evaluate this approach may be more limited by a reduced volume of penetrating trauma.

## 2 Method

### 2.1 Search Strategy

Six databases—Medline (via PubMed), Google Scholar, Scopus, Embase, the International Clinical Trials Registry, and Web of Science—were searched for a 15‐year period dating back from November 2023. Details of the search strategies are shown in Appendix 1. Reference lists from identified results were also searched for results meeting the inclusion criteria. Preferred Reporting Items for Systematic reviews and Meta‐Analyses (PRISMA) [18] was used as the reporting method for this systematic review. The review was registered on the PROSPERO register (CRD42023430786) [19].

### 2.2 Inclusion and Exclusion Criteria

The inclusion criteria were randomized controlled trials, nonrandomized studies, and observational studies of human participants aged ≥ 16 years who had sustained an abdominal stab wound (anterior/posterior/flank/thoracoabdominal) in isolation or as polytrauma. If studies explicitly stated that they contained patients who were < 16 years old, they were excluded unless they contained specific separate data for ≥ 16‐year‐olds that could be extracted—in which case this group was included. Studies that did not specifically report the age range but gave a mean and standard deviation meeting the inclusion criteria were included. Studies had to be conducted in a country defined as upper‐middle‐income, lower‐middle‐income, low‐income, and least developed by the Organization for Economic Co‐operation and Development (2023 classification) [20]. Studies must have been published within 15 years of the search date (November 2023).

Studies had to include a group of patients where SNOM was attempted and needed to state the number of patients in this group who failed this approach and required surgery. We defined attempted SNOM as having started down the path of serial clinical examination or if imaging had been performed and a decision made not to proceed to surgery based on that imaging. The primary outcome for this systematic review was failure of SNOM (need for surgery in a patient initially managed with SNOM); secondary outcomes included mortality, length of stay (LOS), and complications. Studies were included if the primary outcome data could be extracted even if secondary outcomes could not. Non‐English‐language studies were excluded. Case reports and small‐scale case series (containing < 5 patients) were excluded. Studies conducted in upper‐middle‐income, lower‐middle‐income, low‐income, and least developed countries by high‐income countries (e.g., Western military forces in Afghanistan) were excluded based on the centers conducting these studies having equal (or higher) resources than centers in high‐income countries.

### 2.3 Screening and Selection

Abstracts of identified results were screened independently and in duplicate by SM and DB. Disagreements were resolved via discussion/consensus with unresolved disputes being resolved by VK. Remaining results underwent full‐text screening—again independently and in duplicate by SM and DB, with disputes being settled by discussion/consensus and unresolved disputes being settled by VK.

### 2.4 Data Extraction and Quality Assessment

Data extraction (to a preprepared Microsoft Excel spreadsheet) and quality assessment for studies meeting the inclusion criteria were conducted independently and in duplicate by SM and DB, with disputes being resolved by consensus or by VK. Quality assessments for included observational studies were conducted using the JBI checklist for case series tool [21]; quality assessment for randomized studies was conducted using the Cochrane RoB 2 tool [22].

### 2.5 Statistical Analysis

Analysis of data was performed using STATA version 18 [23]. Confidence intervals were calculated using the Clopper–Pearson exact method, and a meta‐analysis of selected outcomes was conducted using the DerSimonian and Laird random‐effects model. Proportions for each study were calculated using Freeman–Tukey double‐arcsine transformations. Heterogeneity was assessed using *I* ^2^, and the risk of publication bias was assessed with a Doi plot and LFK index [24].

## 3 Results

### 3.1 Search Strategy

The searches of Medline, Google Scholar, Scopus, Embase, the International Clinical Trials Registry, and Web of Science identified 448, 641, 401, 755, 42, and 561 results, respectively. Manual searching of reference lists identified a further five results that had the potential to meet the inclusion criteria. After removal of duplicates, 2087 results remained. Screening of titles and abstracts left 286 studies for full‐text assessment. Of these, 252 results were excluded after full‐text assessment. It was not possible to access seven [25, 26, 27, 28, 29, 30, 31] full texts through our institutions' libraries or the British Library. Attempts were made to contact the authors of these seven studies; however, there were no responses. These studies could not, therefore, be included, leaving a total of 27 papers [12, 13, 14, 15, 16, 32, 33, 34, 35, 36, 37, 38, 39, 40, 41, 42, 43, 44, 45, 46, 47, 48, 49, 50, 51, 52, 53] that were initially included in the systematic review (Appendix 2).

**FIGURE 1:**
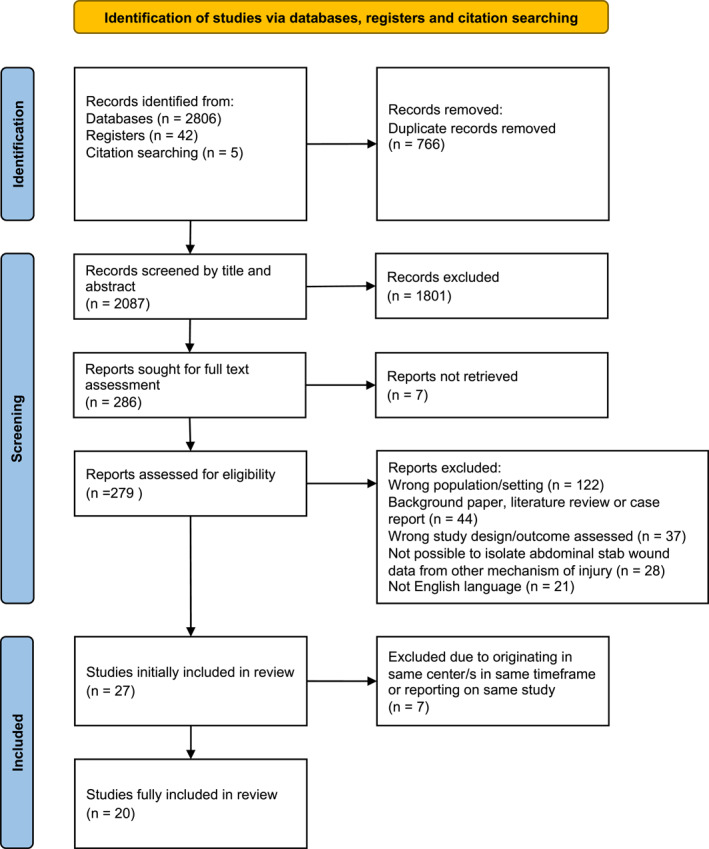
PRISMA chart.

Overall, the 27 papers included 3102 patients for whom SNOM was attempted, with 348 subsequently failing and requiring surgical intervention. However, an analysis of these studies revealed that four papers [44, 45, 48, 49] reported on the same two studies among twelve [12, 34, 35, 43, 44, 45, 46, 47, 48, 49, 51, 52] papers that originated from the same centers with overlap of the reporting periods. To prevent duplication of patients in any pooled results, it was decided that if there was an overlapping reporting period from any center that had published more than one paper, only the most recent study reporting period would be included in further analysis—eliminating seven papers [34, 35, 44, 45, 47, 49, 51]. This resulted in 20 remaining studies [12, 13, 14, 15, 16, 32, 33, 36, 37, 38, 39, 40, 41, 42, 43, 46, 48, 50, 52, 53]—the study characteristics are displayed in Table 1. This left a corrected pooled result of 1505 patients for whom SNOM was attempted, with 245 subsequently failing. The results of this systematic review will only describe the 20 studies included in Table 1 from this point onwards. A PRISMA chart is shown in Figure 1.

**TABLE 1:** Study characteristics.

Name of study	Country of study	Number of centers	Type of center	Type of study	Specific injury included	Age of all included abdominal SW patients	Sex of all included patients	Number of patients with abdominal stab wounds initially managed non operatively	Number of patients who were initially SNOM who failed and required surgery	CT to inform SNOM
Akkoca et al. (2019)	Turkey	1	Training and research hospital	Retrospective evaluation	Anterior abdominal SW	Range 17–90 Mean 32.6 ± 12.7	12F, 89M	34	5	CT tractography, CT abdomen with IV contrast for all included patients.
Breigeiron et al. (2017)	Brazil	1	Referral trauma center	Prospective randomized clinical study	Single‐anterior abdominal SW (could have additional SW elsewhere if not requiring surgery for it)	Median 33.2 SD 13	8F, 58M	66	6	CT group had abdominal CT with IV contrast, serial clinical examination group CT not performed initially but could be performed at clinician’s discretion if patient deteriorated.
Clarke, Allorto and Thomson (2010)	South Africa	3	District, regional and tertiary (level 1 trauma center)	Audit (retrospective)	Anterior abdominal SW	Not reported	Not reported	148	30	Study does not report any CT scans being performed
Clements et al. (2022)	South Africa	1	Level 1 trauma center	Retrospective review	Penetrating abdominal injury (SW and GSW) with renal injury diagnosed on CT or intraoperatively	Median 25 IQR 22–31	2F, 66M	56	4	CT performed at time of admission if it was deemed appropriate
Dayem et al. (2022)	Egypt	1	Teaching hospital	Prospective observational study	Penetrating anterior abdominal SW	Range 16–49 Mean 33 ± 6.8	1F, 76M	64	4	CT tractography, CT abdomen with oral and IV contrast for all SNOM group
Elzeiny and Kassem (2016)	Egypt	1	Teaching hospital	Prospective study	Anterior abdominal SW	Range 18–60 Mean 33.3	12F, 44M	34	8	Patients without immediate indication for surgery underwent abdominal CT scan—if they had a negative FAST scan this was a plain CT if they had a positive FAST scan this was with IV contrast
Fouda, Magdy and Emile (2018)	Egypt	1	Specialized tertiary center	Retrospective chart review	Anterior abdominal SW—deepest part unreachable on clinical examination	Mean 43.9 ± 9.3	0F, 179M	97	9	CT abdomen and pelvis with oral and IV contrast for all SNOM group
Herfatkar et al. (2015)	Iran	1	Trauma referral center	Cross sectional study	Anterior abdominal SW—penetrating into abdominal cavity on local exploration	Range 16–75 Mean 29.9 ± 10.7	9F, 91M	100	8	Study does not report any CT scans being performed
Kaur et al. (2023)	India	1	Level 1 trauma center	Randomized controlled trial	Anterior abdominal SW	Diagnostic laparoscopy group mean 27.3 ± 9.1 Contrast enhanced CT group mean 28.9 ± 9.4	4F, 102M	33	2	CT thorax and abdomen for all patients evaluate for SNOM—IV contrast in all patients, rectal and oral contrast at clinicians discretion, arterial phase in FAST positive patients
Kong et al. (2019)	South Africa	2	Regional and tertiary (level 1 trauma center)	Retrospective review	Anterior abdominal SW with isolated omental evisceration	Mean 27	37F, 368M	181	20	CT not routinely performed
Loulah et al. (2019)	Egypt	1	Teaching hospital	Prospective cross sectional study	Anterior abdominal SW	Range 25–70 Mean 51.9 ± 13.3	11F, 29M	40	4	CT used for confirmation of diagnosis (no CTs reported in study)
Murari et al. (2023)	India	Not reported	Not reported	Prospective study	Penetrating abdominal SW with organ or omentum evisceration	Mean 28.9	2F, 28M	22	11	Not reported
Nwashilli and Egigba (2021)	Nigeria	1	University teaching hospital	Retrospective review	Abdominal SW (anterior, posterior and flanks)	Range 17–50 Mean 30 ± 8.9	4F, 30M	14	0	Unclear—paper states ‘radiological investigations (abdominal ultrasound/abdominal CT scan) are carried out’ but does not provide further figures on which modality was used and how often
Okus et al. (2013)	Turkey	1	Teaching hospital	Retrospective analysis	Abdominal trauma (SW, GSW and blunt trauma)	Information specific to abdominal SW patients not reported—for all included patients (SW/GSW/blunt trauma) Mean 38.6 ± 15.7	Information specific to abdominal SW patients not reported—for all included patients (SW/GSW/blunt trauma) 17F, 98M	42	5	Not reported
Olaogun et al. (2020)	Nigeria	1	Emerging semi‐urban teaching hospital	Prospective descriptive study	All abdominal injuries (SW, GSW, falls, unsafe abortion, and road traffic injury)	Information specific to abdominal SW patients not reported—for all included patients (all penetrating abdominal trauma) Range17‐72 Mean 34.2 ± 10.8 median 34	4F, 42M	5	0	No patient underwent CT as scanner not available at this center
Paydar et al. (2012)	Iran	1	Level 1 trauma center	Quasi randomized study	Anterior abdominal SW	SNOM group Mean 26.4 Operative management group Mean 27.1	10F, 89M	47	6	CT not routinely performed
Paydar, Ravanfar and Shakoori (2014)	Iran	1	Trauma teaching hospital	Prospective study	Anterior abdominal SW	Mean 34.4	8F, 37M	45	27	CT performed if laceration located near solid organ and solid organ injury could not be determined by physical examination
Sarici and Kalayci. (2018)	Turkey	1	Large urban teaching hospital and high volume trauma center	Prospective observational study	Anterior abdominal SW	Range 16–42 Mean 22	24F, 228M	194	86	CT tractography, CT abdomen with oral and IV contrast for all SNOM group
Sarigoz et al. (2019)	Turkey	1	Training and research hospital	Retrospective study	Posterior abdominal and flank SW	Mean 26 ± 8	2F, 23M	25	7	CT tractography, CT abdomen with IV contrast for all SNOM patients.
Wolmarans, Fru and Moeng (2023)	South Africa	1	Level 1 trauma center	Retrospective review	Penetating abdominal injury (SW and GSW)—abdomen defined as between 5th intercostal space, pubic symphysis, inguinal ligaments, iliac crests, and mid scapular lines	Information specific to the included abdominal SW patients not reported—for all included patients (SW and GSW) Range 18–68 Mean 31.5	32F, 405M	258	3	CT abdomen with IV contrast for all patients evaluated for SNOM

### 3.2 Study Characteristics

The included studies took place in South Africa (*n* = 4) [42, 43, 46, 48], Egypt (*n* = 4) [12, 16, 32, 33], Turkey (*n* = 4) [14, 50, 52, 53], Iran (*n* = 3) [38, 39, 40], India (*n* = 2) [36, 37], Brazil (*n* = 1) [15], and Nigeria (*n* = 1) [41]. The majority of included studies were single‐center studies. Two studies were from more than one center [46, 48]. The types of center varied considerably from district hospital level [46] to level 1 trauma center [36, 39, 42, 46, 48]. The majority of studies included patients with anterior abdominal SW. Several reported on other forms of abdominal trauma (with it being possible to extract SW data as per our inclusion criteria). Sarigoz et al. [52] reported on flank and posterior abdomen SW. Nwashilli and Egigba [13] reported on anterior, flank, and posterior abdominal SW, and Wolmarans, Fru, and Moeng [42] reported on penetrating abdominal injuries with the mid‐scapular lines as the borders (thus including the flanks and some posterior abdomen). The study by Kong et al. [48] was specifically investigating omental evisceration, and the study by Clements et al. [43] was focusing on renal injury after abdominal SW. All included studies required patients to be hemodynamically stable without signs of peritonitis to be eligible for consideration for SNOM. The majority of patients included in all studies (not just those undergoing SNOM) were male (pooled total: 91.3% male). The age range of participants (pooled age range from all included studies) was from 16 to 90 years old.

Eight studies [12, 14, 16, 32, 36, 42, 50, 52] used mandatory CT scanning to inform SNOM; three studies [33, 38, 43] conducted CT at clinician discretion. Details of CT protocols/use of contrast are shown in Table 1. The use of FAST scan/ultrasonography was reported by eight studies [12, 13, 32, 33, 36, 38, 41, 46]. It was reported as being a routine part of the management protocol for abdominal SW patients in five of these studies [12, 32, 33, 36, 38]. Interestingly, in two studies, FAST results were used to inform the protocol and/or the type of contrast used for CT scanning [32, 36]. All studies employed serial clinical examination. The timings of these varied considerably among studies. Eleven studies [12, 13, 14, 15, 16, 32, 36, 38, 39, 40, 46] reported the frequency of serial clinical examination. The initial frequency of clinical examination was two hourly (*n* = 4) [12, 14, 38, 39], four hourly (*n* = 5) [16, 32, 36, 40, 46], six hourly (*n* = 1) [15] and twelve hourly (*n* = 1) [13]. The frequency of examinations was reduced as patients progressed successfully through the SNOM observation period in four studies [12, 14, 32, 40]. When the grade of the person performing the clinical examination was reported, it was performed by a reasonably experienced clinician (surgical resident level as a minimum) [36, 39, 40, 46, 48]. The initial frequency of observation of vital signs for SNOM patients was described in six papers [12, 13, 15, 16, 32, 48]. There was continuous monitoring (in a high dependency unit) (*n* = 1) [16], two hourly (*n* = 2) [12, 48], four hourly (*n* = 2) [13, 32], and six hourly (*n* = 1) [15] observations. Again, some studies reported that the frequency of observation of vital signs decreased as patients moved through the SNOM observation period [12, 13, 32]. Seven studies reported carrying out blood tests during the SNOM observation period [12, 13, 14, 16, 38, 39, 40]. Breigeiron et al. explicitly reported not conducting blood tests post‐randomization of patients [15]. The initial frequency of blood tests was two hourly (*n* = 1) [14], four hourly (*n* = 1) [16], six hourly (*n* = 4) [12, 13, 38, 39] and eight hourly (*n* = 1) [40]. The overall period of time beyond which SNOM was considered to have been successful was also quite variable among studies. Five studies considered SNOM successful at 24 h [15, 40, 41, 42, 52], one at 24–36 h [16], three at 24–48 h [36, 46, 50], four at 48 h [14, 32, 37, 48], one at 72 h [12] and one at 72–120 h [33]. Three studies reported the time of reintroduction of oral intake, 24 h (*n* = 2) [38, 53] and 36 h (*n* = 1) [39] but did not report the overall time after which SNOM would be considered successful. In two studies [13, 43], this time period was not reported/was unclear.

### 3.3 Quality Assessment/Risk of Bias

Overall, 17 of the studies [12, 13, 14, 16, 32, 33, 37, 38, 40, 41, 42, 43, 46, 48, 50, 52, 53] were assessed using the JBI checklist for case series tool [21]; assessment of these studies had shown that they fitted the JBI definition of case series and were suitable for assessment with this tool. The remaining studies [15, 36, 39] were randomized or quasi‐randomized, so they were assessed using the Cochrane RoB 2 tool [22]. There was risk of bias (RoB) and quality issues with the majority of the included studies (Tables 2 and 3); however, our assessment is that this is unlikely to have influenced the outcomes. There is considerable methodological and clinical heterogeneity in terms of the use of imaging and the purpose of included studies (as described in the previous section). The method used to select patients for SNOM is likely to have contributed to the chances of success; therefore, this heterogeneity is likely to have contributed to the considerable differences between the failure rates of SNOM between studies. Statistical heterogeneity was high, with an *I* ^2^ of 92.6%.

**TABLE 2:** JBI checklist for case series assessment.

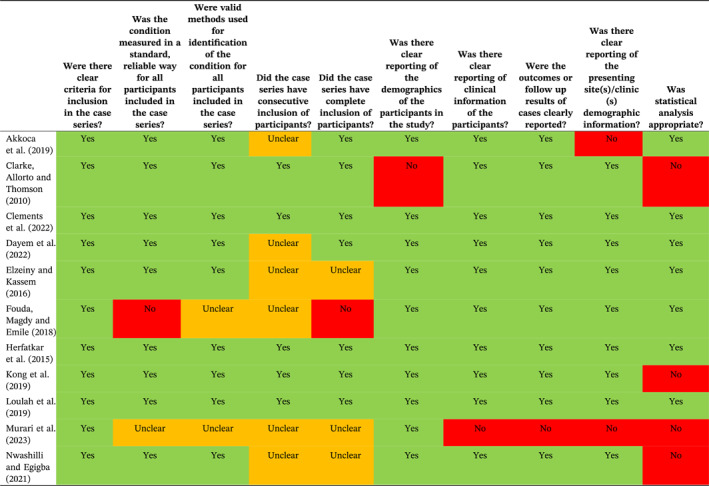
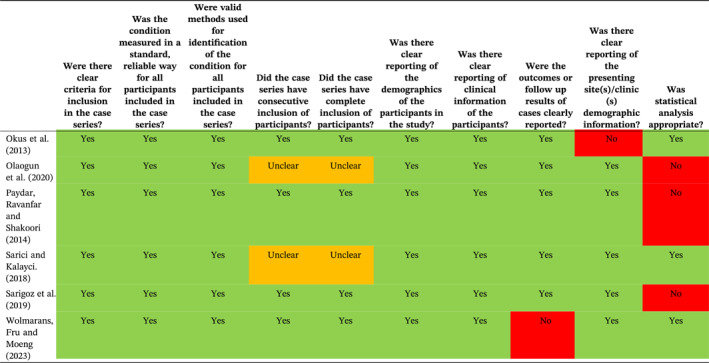

**TABLE 3:** RoB 2 assessment.

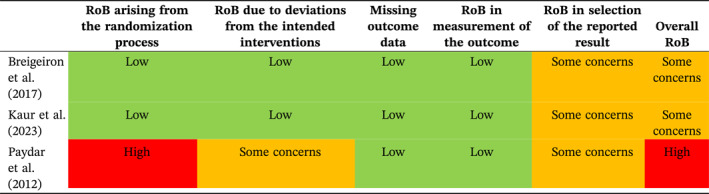

### 3.4 Conflicts of Interest and Publication Bias

**FIGURE 2:**
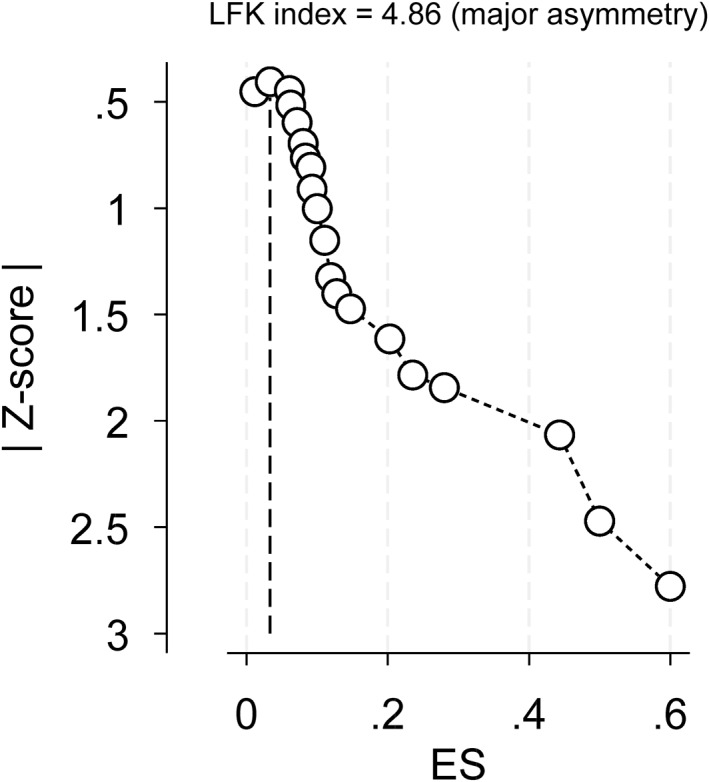
Doi plot and LFK index. Doi plot with LFK index for the included studies in the meta‐analysis for the primary outcome of failed SNOM.

Sixteen of the included studies [12, 13, 14, 15, 16, 33, 36, 37, 41, 42, 43, 46, 48, 50, 52, 53] included conflicts‐of‐interest statements. Fifteen studies [12, 13, 14, 15, 16, 33, 36, 37, 41, 42, 46, 48, 50, 52, 53] had no conflicts of interest to declare, and one study [43] declared that one of the authors had consulted for medical technology companies and was the principal investigator for a trial that was sponsored by a medical technology company. Four studies [32, 38, 39, 40] did not feature any conflicts‐of‐interest statement. On analysis of the declared conflicts of interest, we did not feel that it had any impact on the results reported. A Doi plot for the pooled risk of failure of SNOM of abdominal SW showed major asymmetry, which may indicate a high risk of publication bias; this is displayed in Figure 2.

### 3.5 Outcomes

**FIGURE 3:**
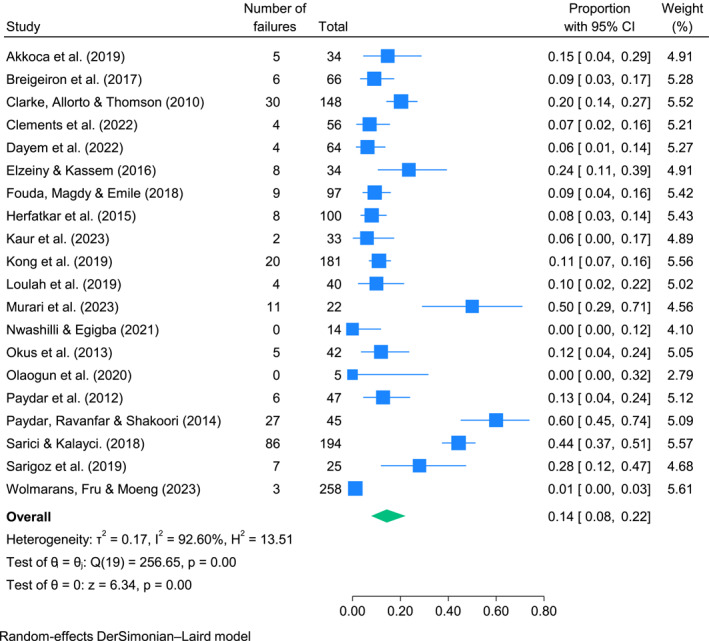
Forest plot for the risk of SNOM failure.

The pooled estimate for the primary outcome (failure of SNOM) for the included 20 studies was 0.14 (95% CI = 0.08–0.22). The *I* ^2^ for the 20 included studies was 92.6%, indicating considerable heterogeneity. A forest plot for the risk of failure of SNOM is shown in Figure 3. Mortality in patients who failed SNOM was specifically reported in 10 studies [13, 33, 36, 39, 40, 41, 43, 46, 50, 53]—in these studies, no patients who failed SNOM went on to die. Of the studies that did not categorically state that they had 0% mortality [12, 14, 15, 16, 32, 37, 38, 42, 48, 52], none mentioned any cases of mortality in SNOM patients. Because of the format of data about LOS, it was not possible to perform a pooled analysis. However, five studies reported LOS in SNOM (on an intention‐to‐treat basis) versus primary operative management [12, 16, 36, 39, 43]. In three of these, LOS was higher in the primary operative management group versus the SNOM group (with *p* values < 0.05) [16, 39, 43]. In one paper, LOS was longer in the SNOM group versus primary operative management (*p* = 0.0248) [12]. Extraction of results from the remaining paper showed a lower median LOS in SNOM patients versus operative management (which, in this study, was diagnostic laparoscopy) (24 h vs. 2.5 days), but a *p* value was not provided, and conflicting numbers for the median LOS in the diagnostic laparoscopy group are described between the text of the paper and one of the tables [36]. A higher mean LOS in mandatory operative management versus SNOM would be an expected finding given that patients would have to recover from surgery. An additional paper reported LOS in successful SNOM versus unsuccessful SNOM with the mean LOS being longer in failed SNOM (2.5 vs. 4.8 days), again with no *p* value reported [38]. Six papers reported the rate of complications (again on an intention‐to‐treat‐type basis) in both an SNOM group (successful or unsuccessful) and primary operative management groups. Two of these had no complications in either group [50, 52]. The remaining four had more complications in the primary operative management groups [12, 16, 36, 43].

## 4 Discussion

The results of this systematic review and meta‐analysis show that SNOM is a safe and feasible management strategy for managing abdominal SW in hemodynamically stable patients without peritonitis. The pooled risk of failure of SNOM from our meta‐analysis is consistent with a very large study by Zafar et al. [54] that found a failure rate of 15.2% in abdominal SW patients (albeit this study was conducted in a high‐income country). The mortality rate shown in this systematic review and meta‐analysis was zero; thus, careful use of SNOM does not appear to increase risk in those who fail the management and require surgery. The included evidence also shows that SNOM can cause a reduction in complications. Using SNOM is a cost‐effective approach, as demonstrated in previous study by Dayananda et al. [6], which has obvious advantages regardless of setting; however, there are also potential disadvantages to using SNOM. In some high‐volume or resource‐stretched settings, the opportunity to intervene surgically if a patient does start deteriorating may be influenced by theater or staff availability. It has been suggested by Olaogun et al. that reduced access to advanced imaging may cause some surgeons to develop a lower threshold for surgical intervention, with this attitude being described as ‘better to look and see, than to wait and be sorry’ [41]. The period of observation beyond which SNOM is considered successful has been demonstrated to be quite variable across the included studies. The experience in our center (Pietermaritzburg Metropolitan Trauma Service) is that most cases that require operative intervention declare themselves within 12 hours of observation, but a subgroup does declare beyond this period [46], although a thorough exploration of the optimum SNOM observation period is beyond the scope of this review.

SNOM of abdominal SW has evolved to incorporate advances in imaging modalities. Initially, this method of managing SW relied solely on clinical examination, but this systematic review has demonstrated that CT scanning has been incorporated successfully by many centers in low‐ and middle‐income countries (despite SNOM relying solely on clinical examination also having been shown to be effective in this systematic review and meta‐analysis and elsewhere) [10]. In trauma systems in high‐income countries where CT scanners are more likely to be co‐located with resus bays, we would suggest that attempts to manage abdominal SW with a conservative approach should incorporate this modality. Evidence from higher‐income countries suggests that some centers have adopted this approach [55], and the World Society of Emergency Surgery recommends it [56]. CT does have limitations in the detection of hollow viscus injury. Wolmarans, Fru, and Moeng (2023) demonstrated that single‐contrast CT had a sensitivity of 95.12% and a specificity of 44.23% in detecting hollow viscus injury in penetrating abdominal trauma. We would agree with their conclusion that CT findings need to be interpreted alongside the clinical picture [42].

Some centers use violation of the anterior fascia on local wound exploration as an indication for either mandatory operative management or for assessment with further diagnostic modalities (such as diagnostic peritoneal lavage or laparoscopy). Mandatory laparotomy based on anterior fascial violation has limitations because most patients will not have visceral injury as demonstrated by Sanei et al. (2013), who found that 82% of patients with anterior abdominal fascial penetration on local wound exploration without peritonitis did not have visceral injury on laparotomy [57]. A potential further evolution in the conservative approach that was identified during our search (but outside of the inclusion criteria for this review and not thoroughly investigated) is the incorporation of diagnostic laparoscopy in decision‐making about the need for laparotomy [58, 59, 60, 61]—in our opinion, this warrants thorough analysis in the form of its own systematic review.

## 5 Limitations

This systematic review has limitations. The majority of included studies are single armed rather than comparative studies, and there are quality assessment issues with all of the included studies. We acknowledge that a broad range of inclusion criteria was used, which was done to avoid excluding potentially relevant studies. Therefore, the majority of the studies are not designed solely to assess SNOM against standard treatment. Well‐designed RCTs of SNOM (guided by clinical observation ± CT imaging or diagnostic laparoscopy) versus primary operative management are warranted. There is considerable heterogeneity among the participants in the included studies, the resources of the center/s involved and the type of SW that patients have sustained. These are major factors that readers should consider when assessing the transportability of the findings to other environments and systems. We made the decision prior to the registration of this systematic review’s protocol to focus on adult patients (≥ 16 years‐old), which led to the exclusion of potentially relevant studies that included a minority of patients under this cutoff. Although we do not believe this has made a substantial change to the results, it reduced the overall number of patients included in the pooled analysis.

## 6 Conclusion

SNOM management of abdominal SW in upper‐middle‐income, lower‐middle‐income, low‐income, and least developed countries is an effective and safe way to manage these injuries. Serial clinical examination and regular observation of vital signs remain the foundation of this management strategy. However, the take‐home message from this systematic review and meta‐analysis is that advances in imaging and other diagnostic modalities are increasingly being successfully incorporated as adjuncts to this management approach. The success of SNOM in upper‐middle‐income, lower‐middle‐income, low‐income, and least developed countries should be considered in decision‐making around management of abdominal SW in higher‐resource settings.

## Author Contributions

**Samuel Moffatt:** conceptualization; data curation; formal analysis; investigation; methodology; project administration; writing – original draft; writing – review & editing. **Daniel Biggs:** data curation; formal analysis; writing – review & editing. **Victor Kong:** conceptualization; data curation; supervision; writing – review & editing. **Damian Clarke:** conceptualization; supervision; writing – review & editing.

## Conflicts of Interest

The authors declare no conflicts of interest.

## Supporting information

Supplementary Material

Supplementary Material

## Data Availability

The authors confirm that the data supporting the findings of this study are available within the article and its Supplementary Materials.
