# Supplementary material for: Selective Nonoperative Management of Abdominal Stab Wounds in Low‐ and Middle‐Income Countries: A Systematic Review and Meta‐Analysis
Source: World J Surg. 2025 Mar 17;49(4):1115–27. doi: 10.1002/wjs.12517 (PMC11994151; doi:10.1002/wjs.12517)
Supplement: Supplementary file 1 — Supplementary Material [file WJS-49-1115-s002.docx]

Appendix 1. Search strategy

| **Medline**  **(via PubMed)** | ((stab) **OR** (stabbing) **OR** (penetrat*) **OR** (knife)) **AND** ((abdo*) **OR** (thoracoabdo*) **OR** (flank)) **AND** ((non-operative) **OR** (nonoperative) **OR** (conservat*) **OR** (selective*)) |
| --- | --- |
| **Google Scholar** | allintitle: conservative **OR** selective **OR** "non operative" **OR** "non operative" **OR** selective **OR** abdo **OR** stab "abdomen **OR** abdominal"    **Date range 2008-2023** |
| **SCOPUS** | TITLE-ABS ( stab **OR** stabbing **OR** penetrat* **OR** knife ) **AND** TITLE-ABS ( abdo* **OR** thoracoabdo* **OR** flank ) **AND** TITLE-ABS ( non-operative **OR** nonoperative **OR** conservat* **OR** selective* ) **AND** PUBYEAR > 2008 AND PUBYEAR < 2024 **AND** ( LIMIT-TO ( SUBJAREA , "MEDI" ) ) |
| **EMBASE** | 1. stab  2. stabbing  3. penetrat*  4. knife  5. abdo*  6. thoracoabdo*  7. flank  8. non-operative  9. nonoperative  10. conservat*  11. selective*  12. 1 **OR** 2 **OR** 3 **OR** 4  13. 5 **OR** 6 OR 7  14. 8 **OR** 9 **OR** 10 **OR** 11  15. 12 **AND** 13 **AND** 14  16. Limit search result from 2008 to 2023 |
| **International Clinical Trials Registery** | (stab **OR** stabbing **OR** penetrat* **OR** knife) **AND** (abdo* **OR** abdomen **OR** flank) |
| **Web of Science** | 1. **((ALL=(stab)) OR ALL=(stabbing)) OR ALL=(penetrat*)**  **2. ((ALL=(abdo*)) OR ALL=(thoracoabdo*)) OR ALL=(flank)**  **3. (((ALL=(non-operative)) OR ALL=(nonoperative)) OR ALL=(conservat*)) OR ALL=(selective*)**  **4. #3 AND #2 AND #1**  **5. Years 2008 - 2023** |
