# Supplementary material for: Selective Nonoperative Management of Abdominal Stab Wounds in Low‐ and Middle‐Income Countries: A Systematic Review and Meta‐Analysis
Source: World J Surg. 2025 Mar 17;49(4):1115–27. doi: 10.1002/wjs.12517 (PMC11994151; doi:10.1002/wjs.12517)
Supplement: Supplementary file 2 — Supplementary Material [file WJS-49-1115-s001.docx]

| **Name of study** | **Country of study** | **Specific hospital or group** | **Dates included in study** |
| --- | --- | --- | --- |
| Breigeiron et al. (2017) | Brazil | Hospital de Pronto Socorro de Porto Alegre | Jul 2011 - Feb 2015 |
| Elzeiny & Kassem (2016) | Egypt | Alexandria Main University Hospital | May 2013 - Oct 2014 |
| Loulah et al. (2019) | Egypt | Damanhour Medical National Institute | Jun 2017 - Feb 2018 |
| Maurice et al. (2021) | Egypt | Kasr Alainy Teaching Hospital | Sept 2017 - Sept 2018 |
| Dayem et al. (2022) | Egypt | Kasr Alainy Teaching Hospital | Aug 2018 - Aug 2020 |
| Elshal et al. (2022) | Egypt | Kasr Alainy Teaching Hospital | Feb 2020 - Aug 2020 |
| Fouda, Magdy & Emile (2018) | Egypt | Mansoura University Emergency Hospital | Jan 2011 - Mar 2016 |
| Kaur et al. (2023) | India | JPN Apex Trauma Centre | May 2019 - Feb 2021 |
| Murari et al. (2023) | India | Not Reported | Not Reported |
| Paydar et al. (2012) | Iran | Nemazee hospital | Aug 2009 - Aug 2010 |
| Herfatkar et al. (2015) | Iran | Poursina Hospital | Sept 2009 - Sept 2012 |
| Paydar, Ravanfar & Shakoori (2014) | Iran | Rajaiee Trauma Center | Sept 2012 - Sept 2013 |
| Olaogun et al. (2020) | Nigeria | Ekiti State University Teaching Hospital | Jan 2015 - Dec 2018 |
| Nwashilli & Egigba (2021) | Nigeria | University of Benin Teaching Hospital | Jan 2014 - Dec 2017 |
| Wolmarans, Fru & Moeng (2023) | South Africa | Charlotte Maxeke Johannesburg Academic Hospital | Aug 2017 - Aug 2019 |
| Clements et al. (2022) | South Africa | Groote Schuur Hospital | Apr 2015 - Jan 2019 |
| Sander et al. (2022) [A prospective audit of 805 consecutive patients with penetrating abdominal trauma: evolving beyond injury mechanism dictating management] | South Africa | Groote Schuur Hospital | May 2015 - Apr 2017 |
| Sander et al. (2022) [Penetrating abdominal trauma in the era of selective conservatism: a prospective cohort study in a level 1 trauma center] | South Africa | Groote Schuur Hospital | May 2015 - Apr 2017 |
| Clarke, Allorto & Thomson (2010) | South Africa | Pietermaritzburg Metropolitan Trauma Service | Jan 2007 - Jan 2009 |
| Kong et al. (2015) | South Africa | Pietermaritzburg Metropolitan Trauma Service | Jan 2008 - Dec 2013 |
| Kong et al. (2019) | South Africa | Pietermaritzburg Metropolitan Trauma Service | Jan 2010 - Jan 2018 |
| Kong et al. (2021) | South Africa | Pietermaritzburg Metropolitan Trauma Service | Jan 2010 - Dec 2017 |
| Akkoca et al. (2019) | Turkey | Dışkapı Yıldırım Beyazıt Research and Training Hospital | 2011 - 2016 |
| Sarici & Kalayci. (2018) | Turkey | Kanuni Sultan Suleyman Training and Research Hospital | Nov 2015 - Sept 2017 |
| Ertan et al. (2015) | Turkey | Kayseri Training and Research Hospital, Kayseri, Turkey | 2012 - 2014 |
| Sarigoz et al. (2019) | Turkey | Kayseri Training and Research Hospital | 2012 - 2017 |
| Okus et al. (2013) | Turkey | Konya Teaching Hospital | Nov 2008 - Jan 2013 |

Appendix 2. Studies initially meeting inclusion criteria
